# Adverse effect profile of trichlormethiazide: a retrospective observational study

**DOI:** 10.1186/1475-2840-10-45

**Published:** 2011-05-23

**Authors:** Yasuo Takahashi, Yayoi Nishida, Tomohiro Nakayama, Satoshi Asai

**Affiliations:** 1 Division of Genomic Epidemiology and Clinical Trials, Advanced Medical Research Center, Nihon University School of Medicine, 30-1 Oyaguchi-Kamimachi, Itabashi-ku, Tokyo 173-8610, Japan; 2 Division of Clinical Trial Management, Advanced Medical Research Center, Nihon University School of Medicine, 30-1 Oyaguchi-Kamimachi, Itabashi-ku, Tokyo 173-8610, Japan; 3 Division of Laboratory Medicine, Department of Pathology and Microbiology, Nihon University School of Medicine, 30-1 Oyaguchi-Kamimachi, Itabashi-ku, Tokyo 173-8610, Japan

## Abstract

**Background:**

Trichlormethiazide, a thiazide diuretic, was introduced in 1960 and remains one of the most frequently used diuretics for treating hypertension in Japan. While numerous clinical trials have indicated important side effects of thiazides, e.g., adverse effects on electrolytes and uric acid, very few data exist on serum electrolyte levels in patients with trichlormethiazide treatment. We performed a retrospective cohort study to assess the adverse effects of trichlormethiazide, focusing on serum electrolyte and uric acid levels.

**Methods:**

We used data from the Clinical Data Warehouse of Nihon University School of Medicine obtained between Nov 1, 2004 and July 31, 2010, to identify cohorts of new trichlormethiazide users (n = 99 for 1 mg, n = 61 for 2 mg daily dosage) and an equal number of non-users (control). We used propensity-score matching to adjust for differences between users and control for each dosage, and compared serum chemical data including serum sodium, potassium, uric acid, creatinine and urea nitrogen. The mean exposure of trichlormethiazide of 1 mg and 2 mg users was 58 days and 64 days, respectively.

**Results:**

The mean age was 66 years, and 55% of trichlormethiazide users of the 1 mg dose were female. In trichlormethiazide users of the 2 mg dose, the mean age was 68 years, and 43% of users were female. There were no statistically significant differences in all covariates (age, sex, comorbid diseases, past drugs, and current antihypertensive drugs) between trichlormethiazide users and controls for both doses. In trichlormethiazide users of the 2 mg dose, the reduction of serum potassium level and the elevation of serum uric acid level were significant compared with control, whereas changes of mean serum sodium, creatinine and urea nitrogen levels were not significant. In trichlormethiazide users of the 1 mg dose, all tests showed no statistically significant change from baseline to during the exposure period in comparison with control.

**Conclusions:**

Our study showed adverse effects of decreased serum potassium and increased serum uric acid with trichlormethiazide treatment, and suggested that a lower dose of trichlormethiazide may minimize these adverse effects. These findings support the current trend in hypertension therapeutics to shift towards lower doses of thiazides.

## Background

Thiazide diuretics have remained important medications for the treatment of hypertension for over 50 years, since they became available in the late 1950s [1,2]. Numerous clinical trials have shown the clinical efficacy of thiazides, as well as a reduction in cardiovascular morbidity and mortality, resulting from their blood pressure (BP)-lowering effect [3-5]. Most complications of thiazide therapy are related to their adverse-effect profile. It is well known that thiazides can reduce the excretion of calcium and uric acid, thereby leading to an increase in their plasma levels, and that these drugs increase potassium and magnesium excretion, potentially leading to hypokalemia and hypomagnesemia [6]. Low doses of thiazides, however, are usually tolerated and have been shown to improve quality-of-life measures [7]. Thiazides are now more commonly used at lower doses to minimize the potential harm of adverse effects. Recent reports thus recommend thiazide diuretics as first-line choices for the treatment of essential hypertension, as monotherapy or in combination with other agents [8,9]. Trichlormethiazide, a thiazide diuretic, was introduced in 1960 and remains one of the most frequently used diuretics for treating hypertension in Japan [10]. While many studies have shown an important side effect of thiazides, i.e., adverse effects on electrolytes, very few data exist on plasma electrolyte levels in patients with trichlormethiazide treatment. To clarify whether adverse changes in plasma electrolyte levels are associated with trichlormethiazide is important for clinical practice. In this study, we examined changes in serum electrolyte levels of new users of generally prescribed doses of trichlormethiazide, of 1 mg or 2 mg per day, and compared them with each respective control (who had not received trichlormethiazide). We also examined changes in serum uric acid levels in addition to serum creatinine and urea nitrogen.

## Methods

### Data source

This was a retrospective database study using the Nihon University School of Medicine (NUSM) Clinical Data Warehouse (CDW), which is a centralized data repository that integrates separate databases, such as an order entry database and a laboratory results database, from the hospital information systems at three hospitals affiliated to NUSM. The prescription database in the CDW contains information from over 0.4 million patients, and prescribing data are linked longitudinally to detailed clinical information such as patient demographics, diagnosis, and laboratory results data.

### Study participants

Patients aged over 20 years who had been treated with antihypertensive agents (thiazide diuretic, β-blocker, calcium channel blocker (CCB), α-blocker, angiotensin-converting enzyme inhibitor (ACEI), angiotensin II receptor blocker (ARB), vasodilator or centrally acting agent) for at least three months between Nov 1, 2004 and July 31, 2010, were identified for the study. Of 28,647 patients who fulfilled the above criteria, we identified 1404 patients with trichlormethiazide treatment. We compared new users of trichlormethiazide (1 mg or 2 mg per day) with a propensity-score matched sample of controls who had not used trichlormethiazide. New use of an antihypertensive agent was defined as prescription of the study drug, with no use of this drug in the 90 days preceding the date this prescription was filled (taken as the index date). We excluded patients who had received a potassium or sodium preparation, insulin, allopurinol, uricosuric drug or diuretic (loop, potassium-sparing or thiazide diuretic except for trichlormethiazide) during the study period. We also excluded patients whose serum creatinine level was more than 2 mg/dl before the index date. Because trichlormethiazide was often combined with other antihypertensive agents, such as a CCB, ARB or ACEI, we focused on combination therapy of trichlormethiazide, and excluded patients who had been treated with trichlormethiazide monotherapy. Consequently, all users of trichlormethiazide in this study had received combination therapy with one or more other antihypertensive agents prior to or on the date trichlormethiazide was initiated. In addition, patients who had received these combined agents during at least three months of trichlormethiazide coadministration were selected. The study cohorts thus included 99 new users of trichlormethiazide (1 mg/day) with 99 matched control patients, and 61 new users of trichlormethiazide (2 mg/day) with 61 matched control patients. The experimental protocol was approved by the Ethical Committee of Nihon University School of Medicine.

### Exposure and measurements

The baseline measurement period (non-exposure period) was defined as the 60 days before the index date of any antihypertensive agent. The exposure period (outcome measurement period) was defined as between 1 and 3 months after the start of trichlormethiazide treatment in the trichlormethiazide cohort. Blood test data (serum sodium, potassium, uric acid, creatinine and urea nitrogen) were collected for each individual at the date nearest the index date in the baseline period, and at the date nearest to three months after the start of trichlormethiazide treatment in the exposure period. In trichlormethiazide users, the lag-time for starting treatment with trichlormethiazide from the start of treatment with other combined antihypertensive agents varied among individuals. Therefore, the exposure period in the control cohort was adjusted according to the matched pair in the trichlormethiazide cohort.

### Data elements

For each individual, we collected information of patient demographics (age and sex), medical history, use of antihypertensive agents or other drugs, and laboratory results. Medical history included disease information of cerebrovascular disease (ICD10 codes, I60-I69), ischemic heart disease (I20-I25), other heart disease (I30-I52), liver disease (K70-K77), kidney disease (N00-N19), gout (M10), thyroid gland disorders (E00-E07), hyperlipidemia (E78.0-E78.5), and diabetes mellitus (E10-E14), which had been diagnosed in the 365 days preceding the first date the prescription of any antihypertensive agent was filled. This medical history also included the binary laboratory results of whether urinary protein was positive or not. We noted current users of antihypertensive agents (β-blocker, CCB, α-blocker, α+β-blocker, ACEI, ARB, or vasodilator), defined as patients who received them after the index date up to their outcome measurement. Past users were defined as patients who received drugs during the 90 days before the index date. We also noted past users of other drugs than antihypertensive agents, including steroids, lipid-lowering drugs, oral antihyperglycemic drugs, thyroid drugs and chemotherapeutic drugs.

### Propensity score matching

We used propensity-score matching to reduce bias by balancing covariates between settings. The propensity score for each subject is obtained by fitting a logistic regression model that includes the predictor variable (i.e., users or non-users) as an outcome and all baseline covariates in Table 1[11]. We used all these observed variables regardless of statistical significance. After the propensity score was constructed, we matched the propensity score of each patient with diabetes and without diabetes (a 1:1 match). A nearest-neighbor-matching algorithm with a "greedy" heuristic was used to match patients and the logit of their propensity score, with matching occurring if the difference in the logits of the propensity scores was less than 0.2 times the standard deviation (SD) of the scores (caliper width) [12]. This caliper of 0.2 SD of the logit of propensity score was suggested by Austin after an extensive simulation study [13]. We compared the prevalence of all baseline covariates after PS matching using a *t*-test for continuous variables and chi-squared tests for categorical data.

**Table 1:** Baseline characteristics after propensity score matching

Characteristic	TCM users (1 mg/day) with matched controls			TCM users (2 mg/day) with matched controls		
	
	Users (n = 99)	Controls (n = 99)	p-value	Users (n = 61)	Controls (n = 61)	p-value
Exposure days (days, mean ± SD)	57.8 ± 14.8	-		63.9 ± 16.4	-	
Age (years, mean ± SD)	65.8 ± 13.5	66.4 ± 11.4	0.7476	67.5 ± 9.9	69.2 ± 11.2	0.3741
Women	54 (55%)	61 (62%)	0.3134	26 (43%)	27 (44%)	0.8551
Medical History						
Diabetes mellitus	54 (55%)	57 (58%)	0.6675	42 (69%)	44 (72%)	0.6914
Cerebrovascular diseases	33 (33%)	41 (41%)	0.2399	17 (28%)	11 (18%)	0.1964
Ischemic heart diseases	19 (19%)	21 (21%)	0.7233	12 (20%)	11 (18%)	0.8169
Other heart disease	42 (42%)	35 (35%)	0.3075	34 (56%)	36 (59%)	0.7143
Liver disease	22 (22%)	24 (24%)	0.7364	27 (44%)	25 (41%)	0.7143
Kidney disease	30 (30%)	28 (28%)	0.7548	21 (34%)	17 (28%)	0.4342
Gout	5 (5%)	5 (5%)	1	1 (2%)	1 (2%)	1
Thyroid disorders	27 (27%)	31 (31%)	0.5322	23 (38%)	25 (41%)	0.7109
Hyperlipidemia	59 (60%)	56 (57%)	0.6657	50 (82%)	52 (85%)	0.6248
COPD	5 (5%)	4 (4%)	0.733	5 (8%)	3 (5%)	0.4645
Proteinuria	30 (30%)	32 (32%)	0.7592	22 (36%)	21 (34%)	0.8497
Past drugs						
Chemotherapeutic drugs	1 (1%)	0 (0%)	0.3161	3 (5%)	3 (5%)	1
Oral antihyperglycemic drugs	10 (10%)	13 (13%)	0.5058	11 (18%)	15 (25%)	0.3765
Lipid-lowering drugs	30 (30%)	22 (22%)	0.1964	26 (43%)	23 (38%)	0.5796
Steroids	10 (10%)	7 (7%)	0.4467	9 (15%)	5 (8%)	0.2559
Thyroid drugs	3 (3%)	4 (4%)	0.7004	0 (0%)	0 (0%)	-
Current antihypertensive drugs						
ARB	70 (71%)	67 (68%)	0.6442	35 (57%)	34 (56%)	0.8551
AECI	21 (21%)	23 (23%)	0.7324	14 (23%)	15 (25%)	0.8316
CCB	81 (82%)	83 (84%)	0.7063	44 (72%)	45 (74%)	0.8385
Beta-blocker	14 (14%)	11 (11%)	0.5209	15 (25%)	13 (21%)	0.6668
Alpha+beta-blocker	6(6%)	6 (6%)	1	3 (5%)	3 (5%)	1
Alpha-blocker	8 (8%)	14 (14%)	0.1748	9 (15%)	8 (13%)	0.7938
Alpha-agonist	0 (0%)	0 (0%)	-	0 (0%)	0 (0%)	-
Vasodilator	0 (0%)	1 (1%)	0.3161	0 (0%)	0 (0%)	-

Data are numbers of individuals (%) unless otherwise stated. Abbreviations: TCM, trichlormethiazide; COPD, chronic obstructive pulmonary disease; ARB, angiotensin II receptor blocker; AECI, angiotensin-converting enzyme inhibitor; CCB, calcium channel blocker.

### Statistical analysis

The *t*-test was used to compare the differences in means between the trichlormethiazide use group and the control group. All reported P values are two-sided, and P values of less than 0.05 were considered to indicate statistical significance. All analyses were performed with SAS software, version 9.2 (SAS Institute, Cary, NC).

## Results

Table 1 shows the characteristics of the trichlormethiazide cohorts of 1 mg and 2 mg daily dosage and corresponding control cohorts, after propensity-score matching. There were no statistically significant differences for all covariates between trichlormethiazide users and controls for both doses. The mean age was 66 years, and 55% of trichlormethiazide users of the 1 mg dose were female. In trichlormethiazide users of the 2 mg dose, the mean age was 68 years, and 43% of users were female. The mean exposure of trichlormethiazide of 1 mg and 2 mg users was 58 days and 64 days, respectively. In trichlormethiazide users of 1 mg and 2 mg doses, 82% and 72% were currently using a CCB, and 71% and 57% an ARB, respectively. These findings suggest that the major counterparts of trichlormethiazide combination therapy were CCBs or/and ARBs.

Table 2 shows the results of laboratory test values at baseline and during the exposure period. There were no statistically significant differences in the mean values for all tests between baseline and during the exposure period in control. In trichlormethiazide users, also, the mean values for all tests, except the following findings, showed no significant change between baseline and during the exposure period; mean serum sodium level during the exposure period was significantly lower than that at baseline in trichlormethiazide users of the 1 mg dose, whereas in trichlormethiazide users of the 2 mg dose, mean serum uric acid level during the exposure period was significantly higher than that at baseline. Serum urea nitrogen level also tended to increase. Mean serum levels for all tests, however, remained within normal limits during the exposure period in both trichlormethiazide users and controls.

**Table 2:** Summary of serum chemical data

	TCM users (1 mg/day) with matched controls			
	
Laboratory Test	Users (N = 99)		Controls (N = 99)	
	Mean ± SE	(95% CI)	Mean ± SE	(95% CI)
Urea nitrogen, mmol/L				
Baseline	5.83 ± 0.18	(5.47, 6.20)	5.54 ± 0.18	(5.18, 5.90)
Exposure	6.10 ± 0.20	(5.72, 6.51)	5.53 ± 0.17	(5.19, 5.87)
Creatinine, μmol/L				
Baseline	71.7 ± 1.9	(67.8, 75.6)	66.3 ± 2.2	(61.9, 70.7)
Exposure	73.2 ± 2.3	(68.6, 77.8)	67.9 ± 2.5	(62.9, 72.9)
Uric acid, μmol/L				
Baseline	337.4 ± 8.9	(319.7, 355.1)	316.0 ± 8.1	(300.0, 332.0)
Exposure	349.6 ± 8.5	(332.7, 366.4)	316.8 ± 7.8	(301.3, 332.2)
Sodium, mmol/L				
Baseline	142.0 ± 0.3	(141.5, 142.5)	141.8 ± 0.2	(141.3, 142.2)
Exposure	141.1 ± 0.3*	(140.5, 141.7)	141.4 ± 0.3	(140.8, 141.9)
Potassium, mmol/L				
Baseline	4.24 ± 0.04	(4.17, 4.32)	4.28 ± 0.04	(4.20, 4.35)
Exposure	4.28 ± 0.04	(4.20, 4.36)	4.31 ± 0.04	(4.23, 4.39)

	TCM users (2 mg/day) with matched controls			
	
Laboratory Test	Users (N = 61)		Controls (N = 61)	
	Mean ± SE	(95% CI)	Mean ± SE	(95% CI)

Urea nitrogen, mmol/L				
Baseline	6.15 ± 0.24	(5.68, 6.62)	5.66 ± 0.21	(5.24, 6.09)
Exposure	6.80 ± 0.27	(6.25, 7.34)	5.85 ± 0.22	(5.41, 6.29)
Creatinine, μmol/L				
Baseline	73.4 ± 3.1	(67.2, 79.6)	68.1 ± 2.2	(63.7, 72.5)
Exposure	77.7 ± 3.5	(70.7, 84.7)	70.3 ± 2.6	(65.1, 75.7)
Uric acid, μmol/L				
Baseline	333.7 ± 11.1	(311.5, 355.8)	310.6 ± 9.3	(291.9, 329.1)
Exposure	381.8 ± 11.0**	(359.7, 403.9)	327.0 ± 12.0	(303.0, 351.1)
Sodium, mmol/L				
Baseline	141.8 ± 0.3	(141.2, 142.3)	141.9 ± 0.3	(141.2, 142.5)
Exposure	141.6 ± 0.3	(141.0, 142.3)	141.6 ± 0.3	(141.0, 142.2)
Potassium, mmol/L				
Baseline	4.27 ± 0.06	(4.16, 4.39)	4.32 ± 0.06	(4.21, 4.44)
Exposure	4.13 ± 0.05	(4.03, 4.23)	4.36 ± 0.05	(4.27, 4.45)

*P < 0.05 vs. baseline. **P < 0.01 vs. baseline. Abbreviations: TCM, trichlormethiazide; SE, standard error; CI, confidence interval.

**Figure 1:**
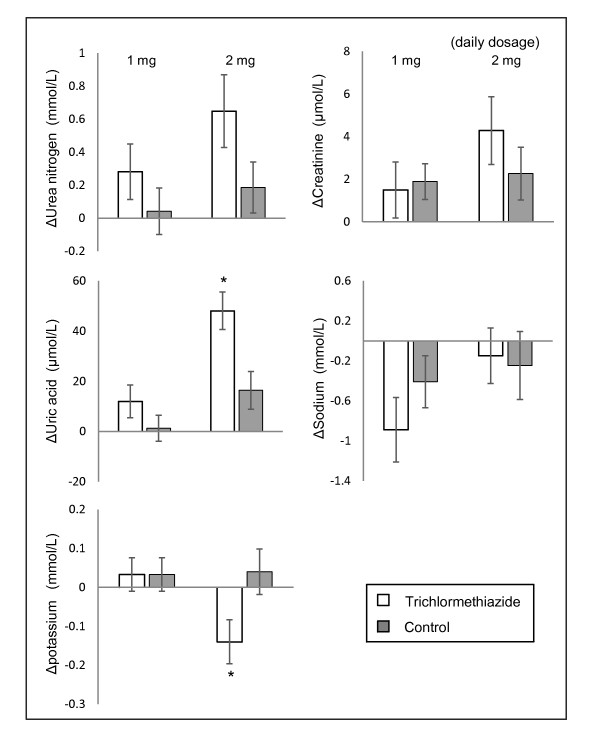
**Mean changes in laboratory test values during the exposure period from baseline**. Data represent mean ± standard error of trichlormethiazide users (open bars) and controls (closed bars). Δ indicates the change in mean serum value during exposure period from baseline. *P < 0.01 vs. Control.

Figure 1 shows the changes in laboratory test mean values during the exposure period compared with baseline. In trichlormethiazide users of the 1 mg dose, all tests showed no statistically significant change from baseline to during the exposure period in comparison with control, whereas in trichlormethiazide users of the 2 mg dose, the reduction of serum potassium level and the elevation of serum uric acid level were significant in comparison with control.

## Discussion

In this study, we found that the reduction of serum potassium level and the elevation of serum uric acid level in trichlormethiazide users of the 2 mg dose were significantly greater than those in trichlormethiazide non-users, but these adverse events did not occur in trichlormethiazide users of the 1 mg dose. Many researchers have reported potential adverse events associated with thiazide therapy, including diuretic-induced hypokalemia and increased serum uric acid [6,14]. Supporting these reports, our study demonstrated some adverse effects, including decreased serum potassium and increased serum uric acid, with trichlormethiazide, the same as with other thiazides. Clinical evidence suggests similar antihypertensive efficacy between low and high doses, with lower complication rates with low doses [15,16]. Franse et al demonstrated a trend suggesting that higher doses of thiazide diuretics correlate with greater reduction in serum potassium level and increased risk of hypokalemia [17]. Hypertension therapeutics in recent years, have thus shifted towards lower doses of thiazides. Because our study was not designed to compare the cohorts with 1 mg and 2 mg doses of trichlormethiazide, we cannot strictly conclude whether the adverse effects on serum potassium and uric acid are dose-dependent or not. However, our study, showing an absence of significant adverse effects in the 1 mg dose of trichlormethiazide users, recommends lower doses for both trichlormethiazide monotherapy and combination therapy, the same as for other thiazides.

A few studies have suggested potential adverse effects of treatment with trichlormethiazide on serum uric acid. The National Intervention Cooperative Study in Elderly Hypertensives (NICS-EH) showed that the increase in serum uric acid level in the trichlormethiazide group was significantly different from that in the nicardipine group [18]. Ishimitsu et al showed that serum uric acid level was significantly higher at the end of combined treatment with trichlormethiazide than following treatment with olmesartan alone [19]. These studies, however, showed no significant reduction of serum potassium level with trichlormethiazide treatment. It is difficult to accurately compare the findings with regard to potassium wasting because of variations in study design and cofounding variables between our study and those trials. This study focused on combination therapy and compared trichlormethiazide users and non-users because a thiazide diuretic is often combined with a CCB, ARB or ACEI in clinical practice in Japan [10]. When the renin-angiotensin-aldosterone system is blocked sufficiently, thiazide diuretics enhance BP lowering and increase the proportion of patients achieving goal BP [6,20]. Combination therapy of these agents with a thiazide diuretic may cancel the adverse effects associated with diuretic therapy, including diuretic-induced hypokalemia, hyperglycemia, and increased serum uric acid [21,22]. In this study, we used propensity-score matching to balance the population of users of other combined antihypertensive agents, a potential confounding variable, between trichlormethiazide users and non-users, and thereby found a significant reduction of serum potassium level in trichlormethiazide users of the 2 mg dose compared with non-users, whereas in the comparison between treatment with 2 mg trichlormethiazide and baseline, serum potassium level tended to decrease, but not significantly. This finding may be biased by various confounders, including other combined agents that would offset the adverse effects of diuretics.

In this study, serum sodium level during treatment with trichlormethiazide at the 1 mg dose was lower than that at baseline, but this negative skew was not seen at the 2 mg dose. This finding may also be confounded by other variables because this result was not compared with that of controls. The discrepancy between 1 mg and 2 mg users of trichlormethiazide could be explained in part by differences in the population studied. Thiazide-induced hyponatremia is less well characterized. It usually occurs within 2 weeks after the initiation of treatment with a thiazide diuretic [23], whereas large trials using long-term thiazides showed a lack of generalized reduction in sodium concentration [24]. Risk factors for the development of hyponatremia include older age, female sex, and low body weight [23,25]. Previously, the NICE-EH trial showed a significant reduction of serum sodium value in the trichlormethiazide group in comparison with the nicardipine group [18]. Trichlormethiazide users in the NICE-EH trial and our study at the 1 mg dose would be similar with respect to unmeasured potential confounders that might differ between 1 mg and 2 mg users of trichlormethiazide. These confounding variables may have a greater impact on the results than trichlormethiazide, because the reduction of serum sodium level in this study was not significant in users compared to non-users of trichlormethiazide. Of the measured variables, the population with other combined antihypertensive agents differed between 1 mg and 2 mg users of trichlormethiazide, and may affect the difference in serum sodium values between the groups. Although it is unclear whether the reduction in sodium concentration in our study was induced by trichlormethiazide or/and other antihypertensive agents, trichlormethiazide is often used for combination therapy with other antihypertensive drugs in real-world practice. Therefore, when prescribing a thiazide, regular checks of electrolyte levels should be performed, especially at baseline and within 2 weeks after initiating therapy.

Our study has some limitations. First, the nature of the observational study involved inherent issues of selection bias and confounding, which are commonly encountered in observational studies estimating the effect of a treatment by comparing outcomes for non-randomized subjects. Although we used rigorous statistical methods to balance potential confounding variables across trichlormethiazide and control users, including propensity-score matching, their ability to control for differences was limited to variables for which they were available or measurable. Nonetheless, the results of well-designed observational studies (with either a cohort or a case-control design) were reported not to systematically overestimate the magnitude of the effects of treatment as compared with those of randomized-controlled trials on the same issue [26]. Second, there is also the possibility of inaccuracy of information in the database (e.g., misclassification of exposure and outcome, ascertainment bias, etc). Prescription claims data and medical records are considered by many to be the gold standard for measuring drug exposure and for capturing intermediate and final outcomes, respectively [27]. NUSM's CDW used in this study may combine the best of both worlds by linking a prescription database to detailed medical information, and is suitable for pharmacoepidemiologic research. Several epidemiological studies examining the effects of antihypertensive drugs on glucose and lipid metabolism using NUSM's CDW have been published to date [28,29].

## Conclusions

In this study, we observed some adverse effects, including decreased serum potassium and increased serum uric acid, with trichlormethiazide, as with other thiazides. These findings support the experience noted in clinical practice that regular checks of electrolyte levels should be performed prior to and after thiazide initiation. In addition, our study suggested that a lower dose of trichlormethiazide may minimize the adverse effects, supporting the current trend in hypertension therapeutics to shift towards lower doses of thiazides. However, the findings of our study, based on a non-randomized design, call for further studies, such as similar analyses of larger and more recent databases, longitudinal studies for a long-term period, and randomized clinical trials for confirmation.

## Competing interests

The authors declare that they have no competing interests.

## Authors' contributions

YT conceived the study and participated in its design. YN performed the statistical analyses. YT drafted the manuscript. YT, TN and MS interpreted the data. All authors have read and approved the final manuscript.

## References

[B1] Freis ED Wanko A Wilson IM Parrish AE Treatment of essential hypertension with chlorothiazide (diuril); its use alone and combined with other antihypertensive agents J Am Med Assoc 1958 166 137 140 13491319 10.1001/jama.1958.02990020025004

[B2] Moser M Macaulay AI Chlorothiazide as an adjunct in the treatment of essential hypertension Am J Cardiol 1959 3 214 219 10.1016/0002-9149(59)90289-9 13626851

[B3] Prevention of stroke by antihypertensive drug treatment in older persons with isolated systolic hypertension. Final results of the Systolic Hypertension in the Elderly Program (SHEP). SHEP Cooperative Research Group JAMA 1991 265 3255 3264 2046107

[B4] Major outcomes in high-risk hypertensive patients randomized to angiotensin-converting enzyme inhibitor or calcium channel blocker vs diuretic: The Antihypertensive and Lipid-Lowering Treatment to Prevent Heart Attack Trial (ALLHAT) JAMA 2002 288 2981 2997 10.1001/jama.288.23.2981 12479763

[B5] Dorsch MP Gillespie BW Erickson SR Bleske BE Weder AB Chlorthalidone reduces cardiovascular events compared with hydrochlorothiazide: a retrospective cohort analysis Hypertension 2011 57 4 689 694 10.1161/HYPERTENSIONAHA.110.161505 21383313

[B6] Ernst ME Moser M Use of diuretics in patients with hypertension N Engl J Med 2009 361 2153 2164 10.1056/NEJMra0907219 19940300

[B7] Grimm RH Jr Grandits GA Cutler JA Stewart AL McDonald RH Svendsen K Prineas RJ Liebson PR Relationships of quality-of-life measures to long-term lifestyle and drug treatment in the Treatment of Mild Hypertension Study Arch Intern Med 1997 157 638 648 10.1001/archinte.157.6.638 9080918

[B8] Chobanian AV Bakris GL Black HR Cushman WC Green LA Izzo JL Jr Jones DW Materson BJ Oparil S Wright JT Jr Roccella EJ Seventh report of the Joint National Committee on Prevention, Detection, Evaluation, and Treatment of High Blood Pressure Hypertension 2003 42 1206 1252 10.1161/01.HYP.0000107251.49515.c2 14656957

[B9] Mancia G De Backer G Dominiczak A Cifkova R Fagard R Germano G Grassi G Heagerty AM Kjeldsen SE Laurent S 2007 Guidelines for the Management of Arterial Hypertension: The Task Force for the Management of Arterial Hypertension of the European Society of Hypertension (ESH) and of the European Society of Cardiology (ESC) J Hypertens 2007 25 1105 1187 10.1097/HJH.0b013e3281fc975a 17563527

[B10] Murai K Obara T Ohkubo T Metoki H Oikawa T Inoue R Komai R Horikawa T Asayama K Kikuya M Current usage of diuretics among hypertensive patients in Japan: the Japan Home versus Office Blood Pressure Measurement Evaluation (J-HOME) study Hypertens Res 2006 29 857 863 10.1291/hypres.29.857 17345785

[B11] Austin PC Chiu M Ko DT Goeree R Tu JV Faries DE, Leon AC, Haro JM Propensity Score Matching for Estimating Treatment Effects Analysis of Observational Health Care Data Using SAS 2010 Obenchain RL: Cary: SAS Press 51 84

[B12] Kosanke J Bergstralh E GMATCH macro for SAS http://mayoresearch.mayo.edu/mayo/research/biostat/upload/gmatch.sas Accessed FEB 1, 2011

[B13] Austin PC The performance of different propensity-score methods for estimating relative risks J Clin Epidemiol 2008 61 537 545 10.1016/j.jclinepi.2007.07.011 18471657

[B14] Neff KM Nawarskas JJ Hydrochlorothiazide versus chlorthalidone in the management of hypertension Cardiol Rev 2010 18 51 56 10.1097/CRD.0b013e3181c61b52 20010338

[B15] Kaplan NM Kaplan NM Treatment of hypertension: drug therapy Clinical Hypertension 2006 9 Philadelphia, PA: Lippincott Williams & Wilkins 213 310

[B16] Bramlage P Hasford J Blood pressure reduction, persistence and costs in the evaluation of antihypertensive drug treatment--a review Cardiovasc Diabetol 2009 8 18 10.1186/1475-2840-8-18 19327149 PMC2669450

[B17] Franse LV Pahor M Di Bari M Somes GW Cushman WC Applegate WB Hypokalemia associated with diuretic use and cardiovascular events in the Systolic Hypertension in the Elderly Program Hypertension 2000 35 1025 1030 10818057 10.1161/01.hyp.35.5.1025

[B18] Randomized double-blind comparison of a calcium antagonist and a diuretic in elderly hypertensives. National Intervention Cooperative Study in Elderly Hypertensives Study Group Hypertension 1999 34 1129 1133 10567194

[B19] Ishimitsu T Numabe A Masuda T Akabane T Okamura A Minami J Matsuoka H Angiotensin-II receptor antagonist combined with calcium channel blocker or diuretic for essential hypertension Hypertens Res 2009 32 962 968 10.1038/hr.2009.133 19696778

[B20] Kalra S Kalra B Agrawal N Combination therapy in hypertension: An update Diabetol Metab Syndr 2010 2 44 10.1186/1758-5996-2-44 20576135 PMC2901246

[B21] Kjeldsen SE Os I Hoieggen A Beckey K Gleim GW Oparil S Fixed-dose combinations in the management of hypertension: defining the place of angiotensin receptor antagonists and hydrochlorothiazide Am J Cardiovasc Drugs 2005 5 17 22 10.2165/00129784-200505010-00003 15631534

[B22] Izzo JL Jr Neutel JM Silfani T Dubiel R Walker F Titration of HCTZ to 50 mg daily in individuals with stage 2 systolic hypertension pretreated with an angiotensin receptor blocker J Clin Hypertens (Greenwich) 2007 9 45 48 10.1111/j.1524-6175.2007.05714.x PMC8110172 17215658

[B23] Clayton JA Rodgers S Blakey J Avery A Hall IP Thiazide diuretic prescription and electrolyte abnormalities in primary care Br J Clin Pharmacol 2006 61 87 95 10.1111/j.1365-2125.2005.02531.x 16390355 PMC1884982

[B24] Elliott WJ Weber RR Murphy MB A double-blind, randomized, placebo-controlled comparison of the metabolic effects of low-dose hydrochlorothiazide and indapamide J Clin Pharmacol 1991 31 751 757 1880234 10.1002/j.1552-4604.1991.tb03772.x

[B25] Chow KM Szeto CC Wong TY Leung CB Li PK Risk factors for thiazide-induced hyponatraemia QJM 2003 96 911 917 10.1093/qjmed/hcg157 14631057

[B26] Concato J Shah N Horwitz RI Randomized, controlled trials, observational studies, and the hierarchy of research designs N Engl J Med 2000 342 1887 1892 10.1056/NEJM200006223422507 10861325 PMC1557642

[B27] Cox E Martin BC Van Staa T Garbe E Siebert U Johnson ML Good research practices for comparative effectiveness research: approaches to mitigate bias and confounding in the design of nonrandomized studies of treatment effects using secondary data sources: the International Society for Pharmacoeconomics and Outcomes Research Good Research Practices for Retrospective Database Analysis Task Force Report--Part II Value Health 2009 12 1053 1061 10.1111/j.1524-4733.2009.00601.x 19744292

[B28] Nishida Y Takahashi Y Nakayama T Soma M Kitamura N Asai S Effect of candesartan monotherapy on lipid metabolism in patients with hypertension: a retrospective longitudinal survey using data from electronic medical records Cardiovasc Diabetol 2010 9 38 10.1186/1475-2840-9-38 20712859 PMC2933671

[B29] Kitamura N Takahashi Y Yamadate S Asai S Angiotensin II receptor blockers decreased blood glucose levels: a longitudinal survey using data from electronic medical records Cardiovasc Diabetol 2007 6 26 10.1186/1475-2840-6-26 17903269 PMC2098751

